# Tendon 3D Scaffolds Establish a Tailored Microenvironment Instructing Paracrine Mediated Regenerative Amniotic Epithelial Stem Cells Potential

**DOI:** 10.3390/biomedicines10102578

**Published:** 2022-10-14

**Authors:** Valentina Russo, Mohammad El Khatib, Giuseppe Prencipe, Annunziata Mauro, Oriana Di Giacinto, Arlette A. Haidar-Montes, Fanny Pulcini, Beatrice Dufrusine, Adrián Cerveró-Varona, Melisa Faydaver, Chiara Di Berardino, Enrico Dainese, Paolo Berardinelli, Matthias Schnabelrauch, Barbara Barboni

**Affiliations:** 1 Unit of Basic and Applied Sciences, Faculty of Biosciences and Agro-Food and Environmental Technologies, University of Teramo, 64100 Teramo, Italy; 2 Department of Biotechnological and Applied Clinical Sciences, University of L’Aquila, 67100 L’Aquila, Italy; 3 Unit of Biochemistry and Molecular Biology, Faculty of Bioscience and Technology for Food Agriculture and Environment, University of Teramo, 64100 Teramo, Italy; 4 Department of Biomaterials, INNOVENT e. V., 07745 Jena, Germany

**Keywords:** tendon, biomimetic, scaffold, amniotic epithelial stem cells, tenodifferentiation, immunomodulation, immunoengineering, YAP, mechanotransduction, regenerative medicine

## Abstract

Tendon tissue engineering aims to develop effective implantable scaffolds, with ideally the native tissue’s characteristics, able to drive tissue regeneration. This research focused on fabricating tendon-like PLGA 3D biomimetic scaffolds with highly aligned fibers and verifying their influence on the biological potential of amniotic epithelial stem cells (AECs), in terms of tenodifferentiation and immunomodulation, with respect to fleeces. The produced 3D scaffolds better resemble native tendon tissue, both macroscopically, microscopically, and biomechanically. From a biological point of view, these constructs were able to instruct AECs genotypically and phenotypically. In fact, cells engineered on 3D scaffolds acquired an elongated tenocyte-like morphology; this was different from control AECs, which retained their polygonal morphology. The boosted AECs tenodifferentiation by 3D scaffolds was confirmed by the upregulation of tendon-related genes (*SCX*, *COL1* and *TNMD*) and TNMD protein expression. The produced constructs also prompted AECs’ immunomodulatory potential, both at the gene and paracrine level. This enhanced immunomodulatory profile was confirmed by a greater stimulatory effect on THP-1-activated macrophages. These biological effects have been related to the mechanotransducer YAP activation evidenced by its nuclear translocation. Overall, these results support the biomimicry of PLGA 3D scaffolds, revealing that not only fiber alignment but also scaffold topology provide an in vitro favorable tenodifferentiative and immunomodulatory microenvironment for AECs that could potentially stimulate tendon regeneration.

## 1. Introduction

Tendinopathies and tendon ruptures represent highly prevalent musculoskeletal disorders in need of being satisfactorily solved, both in humans and animals. Current treatments for these kinds of pathologies have a limited success as a consequence of the absence of valid therapeutic, rehabilitative and diagnostic approaches [1]. Due to the increasing life expectancy, the incidence of tendon disorders progressively rises hence generating a dramatic socio-economic impact [2]. Thus, societal and medical needs place tendinopathies and tendon ruptures at the frontier of advanced responses to health challenges and sectoral policy targets.

Tissue engineering (TE) is considered a promising solution for the regeneration of damaged or diseased tissues [3]. To date, tendon TE approaches adopted so far include decellularized tissues, scaffolds from natural and synthetic origins, growth factors utilization and stem cell-based therapies, or a combination of these strategies [4].

The key role of a scaffold applied in tendon TE consists in providing transient structural and mechanical support to improve tendon tissue healing. Indeed, scaffolds must possess enough mechanical properties, since they play an important role in enabling stress transfer and load bearing during the early phase of regeneration [5,6]. Moreover, its topology, surface topography and chemistry should generate a specific microenvironment able to guide the cell behavior. Various techniques were adopted to fabricate scaffolds for tendon TE, amongst them electrospinning represents the technique of choice due to its ability to produce fibrous matrices able to mimic a natural tendon extracellular matrix (ECM) [7,8].

Poly(lactic-co-glycolic) acid (PLGA) copolymer represents one of the optimum candidates for tendon TE applications due to its high biocompatibility, controllable degradability and sufficient mechanical properties [9,10,11]. It has previously demonstrated its biocompatibility with amniotic epithelial cells (AECs) [12], a promising stem cell source that has shown a great tenodifferentiative potential and high immunomodulatory properties both in vitro [13,14,15,16,17,18,19] and in vivo [20,21,22,23]. Recently, it has been demonstrated that electrospun PLGA fleeces, fabricated with highly aligned topography and fiber diameter size, similar to tendon fibers, exhibited a high teno-inductive potential on AECs. The produced fleeces induced on these stem cells sources an early epithelial–mesenchymal transition (EMT), followed by a trans-differentiation towards the tenogenic lineage within 24 h, without adding any teno-inductive differentiative factors. Furthermore, these fleeces also enhanced AECs immunomodulatory properties [15,16,17]. In this context, topographical cues of certain materials have been shown to influence the tenodifferentiation [15,16,17,24,25] and immunomodulatory properties of the seeded cells and/or of the host tissue, especially in the first 6–48 h after initial contact between scaffold–host [26]. Accordingly, the “immunoengineering” discipline has emerged with the aim of fabricating scaffolds with immunomodulatory properties to improve the interactions between the implanted scaffolds and the host immune system, thus enhancing the regenerative process [26]. Moreover, it has been demonstrated that electrospun scaffolds can influence the immunomodulatory properties of stem cells by modulating the gene and protein expression of cytokines towards an anti-inflammatory phenotype rather than a pro-inflammatory one [6,26,27,28]. It is hypothesized also that these biological effects are activated via mechanotransduction pathways [24,27,29], amongst which Yorkie homologues YAP (Yes-associated protein) represents a key player [26,27,29,30]. Indeed, YAP is a mechanotransducer implicated in the regulation of stem cells fate [30], in particular in their tenogenic differentiation [29] and immunomodulation [27], as it belongs to the mediators connecting mechanical stimuli, amongst which is fiber topography, received by the cytoskeleton and the corresponding cell response [24,26,27]. Since the activation of YAP results in its translocation and accumulation in the nucleus, a significant layer of YAP regulation occurs at the subcellular distribution level and a rise in its nuclear localization suggests cells’ structure and function stimulation [26].

Within this frame of reference, 3D scaffolds with aligned fibers could be able to boost, mimicking tendon topology, not only the tenodifferentiative potential of stem cells, but also their immunomodulatory functions, and thus potentially contribute to an efficacious tendon regeneration.

Based on these requirements, in the present research, 3D fibrous scaffolds that closely mimic the macroscopic, microscopic architecture and the mechanical properties of native tendons were produced by rolling up [31,32,33] the previously fabricated electrospun highly aligned PLGA fleeces [15,16,17]. Then, it was then verified if the effect of biophysical cues, in terms of fiber alignment and construct topology (i.e., 3D scaffolds vs. fleeces), were able to generate a tailored microenvironment able to guide AECs behavior through cell–material interaction. In particular, the influence of electrospun PLGA constructs on engineered AECs was investigated in terms of tenodifferentiation, analyzing Scleraxis (*SCX*), collagen type I (*COL1*), and tenomodulin (*TNMD*) genes and TNMD protein, and immunomodulation examining interleukin-10 (*IL-10)* and interleukin-12 (*IL-12)* genes. Moreover, the immunomodulatory paracrine potential of engineered cells was evaluated to verify the secretion in the conditioned medium (CM) of immunomodulatory molecules and its biological effect on activated THP-1 macrophages cell line. Finally, the involvement of the mechanotransducer YAP activation was verified in the engineered cells. This study demonstrated that the design of 3D tendon biomimetic scaffolds is able to instruct and modulate AECs’ biological and paracrine signaling, offering a promising strategy for tendon regenerative therapy.

## 2. Materials and Methods

### 2.1. Materials

Poly(lactide-co-glycolide) (PLGA, PLG8523) with an inherent viscosity midpoint of 2.3 dL/g and a lactide to glycolide ratio of 85:15 was purchased from PURASORB^®^ (Corbion Purac, Gorinchem, The Netherlands). Gel permeation chromatography (GPC) was used to determine the PLGA molecular weight (Mw = 258,000 g/mol) after dissolving the polymer in chloroform using polystyrene as external standard. Hexafluoro-2-propanol (HFIP) (99%) was purchased from Apollo Scientific, Manchester, UK. Chloroform used as eluent for GPC analysis was obtained from Fisher Scientific, Schwerte, Germany. All other chemicals and solvents were of analytical grade and utilized as received.

### 2.2. Electrospinning and Fabrication of PLGA Fleeces and 3D Scaffolds

PLGA fleeces were fabricated as previously reported [16,17,34] using a commercial E-Spintronic electrospinning apparatus (Erich Huber, Gerlinden, Germany), equipped with a controllable climate machine and metallic cylindrical drum rotating collector (diameter = 120 mm and circumference = 380 mm). In detail, 12% wt/wt PLGA solution was prepared in HFIP and loaded into a polypropylene syringe of 5 mL connected to a stainless-steel needle (inner diameter = 0.8 mm) with a 35 cm PTFE tube (Intra Special Catheters, Rehlingen-Siersburg, Germany). The electrospinning process has been conducted under a temperature of 22.5 °C and a relative humidity 65%. The polymer solution was electrospun according to the following conditions: applied voltage = 33 kV and flow rate = 0.25 mL/h. The PLGA fleeces were collected on baking paper and put on the rotating collector set at a high-speed of 1000 rpm, to obtain fibers with highly aligned topography, situated 20 cm from the syringe tip. The resulting PLGA fleeces were obtained by electrospinning 250 µL of PLGA solution. Rectangular pieces (10 mm × 7 mm) [16,17,34] were obtained from the PLGA fleeces with aligned microfibers and the width of the strips was rolled up manually to obtain 3D tendon-like scaffolds (3D scaffolds).

#### 2.2.1. Imaging and Morphological Analysis of PLGA Fleeces and 3D Scaffolds

Field-Emission Scanning Electron Microscope (FE-SEM) (Supra 55 VP SEM, Carl Zeiss AG, Jena, Germany) observations were conducted using an accelerating voltage of 5 kV, on samples after being coated in gold using a sputter coater. The average fiber diameter size of the fabricated fleeces and 3D scaffolds was calculated from the SEM images by measuring approximately 100 fibers, randomly chosen, using imageJ software (*n* = 3 fleeces and 3D scaffolds). Fiber orientation was assessed using Directionality plugin in the imageJ software (*n* = 3 fleeces and 3D scaffold). This plugin divides the acquired images into square pieces and computes their Fourier power spectra that allow for the generation of statistics based on the highest peak found, characterized by direction (the center of the Gaussian), dispersion (the standard deviation of the Gaussian), and goodness (the goodness of the fit, 1 is good and 0 is bad).

#### 2.2.2. Mechanical Characterization of PLGA Fleeces and 3D Scaffolds

To evaluate the changes in mechanical properties between electrospun PLGA fleeces (5 cm × 2 cm) and 3D scaffolds (5 cm × 550 µm), destructive tensile tests were performed on the different samples under dry conditions. To determine the cross-sectional area, the thickness of the fleece and the diameter of each scaffold were measured using a digital micrometer. The mechanical tests (*n* = 5 samples per group) were carried out with a Texture Analyzer TA.XT2i (Stable Micro Systems, Godalming, UK) with a 5 kg load of cells. The length for the tested materials was set at 50 mm. Two clamps of the tester were used to fix the two extremities of each sample, before the test was started using a stretch speed of 1 mm/min. The stretch stops automatically once the sample is totally broken. The obtained results were presented as maximum load, elongation at break, ultimate tensile strength, and Young’s Modulus, calculated from the stress–strain curves of each sample.

### 2.3. Biological Evaluation of PLGA Fleeces and 3D Scaffolds on AECs

#### 2.3.1. Ethic Statement

The cells employed in this study were harvested from the amniotic membranes of slaughtered Appenninica breed sheep, regarded as waste reproductive tissues derived from animals slaughtered for food purposes. Therefore, there is no need for an ethical declaration.

#### 2.3.2. Isolation of Ovine AECs

As previously stated by Barboni et al. [35], fetuses at around 2–3 months of pregnancy were employed to harvest amniotic membranes and thereby isolate AECs. The cells were collected from the epithelial layer of the amniotic membrane using an enzymatic digestion procedure with 0.25% Trypsin-EDTA (Sigma Chemical, St. Louis, MO, USA). The enzymatic activity was blocked by adding 10% of Fetal Bovine Serum (FBS) to the cell suspension, which was then filtered using a 40 µm cell filter. After centrifugation, cells were counted with vital trypan blue stain using LUNA-II™ Automated Cell Counter (Logos Biosystems Inc., Gyeonggi-do, Korea). AECs were then cultivated at a density of 3 × 10^3^ cells/cm^2^ on Petri dishes in a culture medium made of α-Minimum Essential Medium Eagle (α-MEM), supplemented with 20% FBS, 1% ultraglutamine, 1% amphotericin, and 1% penicillin/streptomycin, and incubated at 38 °C with 5% CO_2_. When the AECs reached 70% confluence, they were detached with 0.05% Trypsin-EDTA and used for further experiments. Before being used, AECs were characterized for their negativity of hemopoietic markers (CD14, CD58, CD31, and CD45), positivity for both surface adhesion molecules (CD29, CD49f, and CD166) and stemness markers (TERT, SOX2, OCT4, and NANOG), low expression for MHC class I molecules, and the absence of MHC class II (HLA-DR) antigens, as described in previous reports [13,20,35,36]. Moreover, freshly isolated AECs were tested for their negativity to tendon-related genes markers: *SCX*, *COL1*, and *TNMD* [20].

#### 2.3.3. PLGA Constructs Sterilization and Conditioning

Before being used for biological experiments, PLGA fleeces and 3D scaffolds were sterilized and conditioned as detailed previously [12,15,16,17]. Briefly, the rectangular PLGA fleeces and 3D scaffolds were sterilized in 70% ethanol (EtOH) prepared in 0.9% NaCl/distilled water (di-H_2_O) for 10 s. Afterward, both PLGA fleeces and 3D scaffolds were hydrated with sterile phosphate buffer saline (PBS). Finally, both scaffolds were incubated for 1 h at 38 °C with 5% CO_2_ in a cell culture growth medium (GM) composed of α-MEM supplemented with 10% FBS, 1% ultraglutamine, 1% amphotericin, and 1% penicillin/streptomycin.

#### 2.3.4. Cell Seeding on PLGA Fleeces and 3D Scaffolds

According to preliminary studies [15,16,17], AECs were seeded on the PLGA fleeces and 3D scaffolds at the concentration of 0.05 × 10^6^ cells for 1 h in the incubator at 38 °C with 5% CO_2_. Afterwards, 1 mL of GM was added to each sample and cultured at the same condition for 48 h. AECs with the same concentration, cultured on 12-well plates, were used as control (CTR).

#### 2.3.5. Immunofluorescence Analyses on Seeded AECs

CTR and AECs seeded on PLGA fleeces and 3D scaffolds after 48 h of culture were fixed in 4% paraformaldehyde for 15 min and then processed for immunofluorescences (IF) analyses to evaluate:

- Cells morphology by calculating the cellular nuclei aspect ratio.
- Tenogenic differentiation by detecting TNMD, a tendon-related late marker expression.
- Mechano-mediated signal activation of by detecting YAP marker’ cell localization.

The changes in AECs’ morphology within CTR, PLGA fleeces and 3D scaffolds were analyzed as previously reported [37], by calculating the cellular nuclei aspect ratio to discriminate between ovoid and elongated cells. The cellular aspect ratio was measured using ImageJ software (ImageJ 1.53k, NIH, Bethesda, MD, USA) by dividing the cell nuclei width by its length. The nuclei aspect ratio was defined as the ratio of the cells’ breadth to its length. Cells with ratio < 0.5 were considered elongated, while the ratio for ovoid cells was >0.5. Indeed, the lower the ratio, the more is the cell elongated. Three replicates were considered for each experiment.

For TNMD and YAP1 protein detection, the samples (*n* = 3 for each type) were permeabilized in PBS/Triton X-100 0.1% for 5 min at room temperature (RT). After non-specific binding blocking with PBS/BSA 1% for 1 h at RT, TNMD primary antibody (Abcam, Cambridge, United Kingdom) diluted in PBS/BSA 1% (1:100), and YAP1 primary antibody (Sigma-Aldrich, St. Louis, MO, USA) diluted in PBS/BSA 1%—Tween 20 0.05% (1:200) were incubated overnight at 4 °C. Anti-rabbit Alexa Fluor 488 secondary antibody (Molecular Probes, Göteborg, Sweden), diluted in PBS/BSA 1% (1:500) for 1 h at RT, was used for antigen retrieval. Nuclear counterstaining was obtained with DAPI (Vectastain, 1:2000 dilution in PBS) for 15 min at RT or Propidium Iodide (PI) (Sigma-Aldrich, St. Louis, MO, USA, 1:1000 dilution in PBS) for 5 min at RT. Negative control of the reactions was performed by omitting primary antibodies. For TNMD images acquisition, it was utilized by an Axioskop2 Plus incident-light fluorescence microscope (Carl Zeiss, Jena, Germany), equipped with a CCD camera (Axiovision Cam; Carl Zeiss) with a resolution of 1300 × 1030 pixels and connected to a computer workstation provided with an interactive and automatic image analyzer (Axiovision, Carl Zeiss). For YAP1 images acquisition, a Nikon Ar1 laser confocal scanning microscope (Nikon, Düsseldorf, Germany) equipped with the NIS-Element software 4.40 (Nikon, Düsseldorf, Germany), using Plan Fluor 4× and 10× and Plan Apo λ 20× objectives (numerical aperture 0.13, 0.3 and 0.75, respectively, zoom 1.00×, refractive index 1.0, and pinhole size 42.1, 35.8 and 26.8 µm, respectively) were used. The used channels were as follow:

Channel 1: FITC; λexc = 488 nm; λem = 525 nm.

Channel 2: TRITC; λexc = 561.5 nm; λem = 595/50 nm.

#### 2.3.6. Reverse Transcription and Real Time Polymerase Chain Reaction (RT-PCR)

The reverse transcriptase quantitative real-time polymerase chain reaction (RT-qPCR) method was utilized for the comparison of teno-inductive (*TNMD, SCX* and *COL1*)- and immunomodulatory (*IL-10* and *IL-12*)-related genes in CTR and AECs seeded onto PLGA fleeces and 3D scaffolds after 48 h culture. According to previously published reports [15,16], total RNA was extracted by Total RNA Purification Kit (Norgen Biotek Corp., Thorold, ON, Canada) according to the manufacturer’s instructions. In a final volume of 20 µL, one µg of total RNA from each sample was used for cDNA synthesis, employing a reverse transcriptase reaction with Random Hexamer Primers and Tetro Reverse Transcriptase (Bioline, Germany). The SensiFAST TM SYBR Lo-ROX Kit (Bioline, Germany) was utilized to perform real time qPCR analysis using the primer sequences for the interested genes (Table 1) as recommended by the manufacturer. The reaction was performed using a 7500 Fast Real-Time PCR System (Life Technologies, Waltham, MA, USA), which consisted in a two-step cycling protocol of 40 cycles (10 s at 95 °C for denaturation and 30 s at 60 °C for annealing/extension), followed by melt profile analysis (7500 Software version 2.3, Life Technologies, Waltham, MA, USA). The analysis of each gene expression was conducted in triplicate per experiment and for each biological replicate. GAPDH endogenous reference gene was used to normalize the obtained results. The comparative Ct (∆∆Ct) method was applied to assess the relative gene expression ratio (2^−∆∆Ct^).

#### 2.3.7. Conditioned Medium (CM) Collection and Their Immunomodulatory Properties Evaluation

A membrane array (Human Inflammation Antibody Array, Abcam, ab134003), designed to identify 40 distinct immunomodulatory molecules, was used to analyze CM collected from AECs CTR or seeded PLGA fleeces and 3D scaffold. After 48 h of culture, each cell culture condition was replaced with serum-free media for 24 h. At the end of the culture, CM was collected, centrifuged to remove any cell debris, and cryopreserved at −80 °C up until the membrane analysis. Serum-free media alone were used as the negative control for the array assay. According to the manufacturer’s instructions, after blocking with buffer solution for 1 h at room temperature, the array membranes were incubated overnight at 4 °C on a rocking platform with 2 mL of CM from AECs CTR or seeded on PLGA fleeces, or 3D scaffolds. Succeeding the washing steps, the membranes were incubated for 2 h at room temperature (RT) with Biotinylated Antibody Cocktail and then for 2 h with HRP-Conjugated Streptavidin. The membranes were visualized using the included chemiluminescent detection solutions. A BioRad ChemiDoc XRS-plus imaging system (Bio-Rad Laboratories, Milan, Italy) was used to capture the chemiluminescent signal. Image Lab software was used to perform densitometric analysis (Image Lab 6.1, Bio-Rad Laboratories, CA, USA), determining the pixel density of each spot by subtracting the background. The signal density of an individual spot was normalized using the signal of positive control detected within each analyzed membrane [38]. The obtained results were compared by semi-quantitative densitometry of mean pixel density.

#### 2.3.8. Western Blot Analysis

A Western Blot (WB) investigation was carried out to evaluate TGF-β1 protein content in samples’ CM. In detail, equal volume amount (30 µL) of CM collected from CTR, and cells seeded on PLGA fleeces and 3D scaffolds, were processed for WB as previously described [38]. The total protein amount in CM has been normalized on a Ponceau S stain, according to Sander et al. [39]. TGF-β1 expression was detected using a specific primary antibody (Abcam, ab27969, Milan, Italy) diluted (1:250) in tris-buffered saline (TBS) solution, and incubated overnight at 4 °C. Finally, the WB membranes were incubated for 1 h at RT with an anti-mouse secondary antibody conjugated to horseradish peroxidase (HRP) (Santa Cruz, sc-516102, Heidelberg, Germany) diluted in TBS (1:10,000). The ECL substrate was used to visualize the target protein (LiteAblot PLUS, Euroclone, Milan, Italy) and Azure’s 400 detected the chemiluminescent signal (Azure Biosystems, c400, Sierra Ct, Dublin, CA, USA). Image J blot analyzer software was employed for the densitometric analysis (ImageJ 1.53k, NIH, Bethesda, MD, USA).

#### 2.3.9. CM Biological Effect on THP-1 Cells

CM biological effect on activated human macrophages was performed by assessing the IL-6 release through an ELISA assay. In detail, the human THP-1 cells were maintained in RPMI 1640 medium containing glutamine and supplemented with 10% FBS and 1 mM sodium pyruvate (Gibco; Thermo Fisher Scientific, Inc., Waltham, MA, USA). Afterwards, monocytes were differentiated into macrophages, as previously described [40]. THP-1-derived macrophages were treated with the CM diluted 1:1, with complete RPMI medium for 24 h. Thereafter, the supernatants were collected, centrifugated at 3000× *g* for 10 min at 4 °C, filtered using a 0.2 µm Ministart sterile filter (Sartorius, Varedo, Italy), and stored at −80 °C until usage. These cellular supernatants were used to quantify the concentrations of released IL-6 using the Human IL-6 Uncoated Invitrogen ELISA Kit assay (ThermoFisher, San Diego, CA, USA) according to the manufacturer’s directions. The plates were read at 450 nm and the sensitivity of the used ELISA assay was in the range 2–200 pg/mL.

### 2.4. Statistical Analysis

At least three replicates derived from three different PLGA fleeces and 3D scaffolds were used to assess their ultrastructure and mechanical properties. Biological experiment analyses with AECs and their CM were performed by using cells isolated from the amnia obtained from at least three different animals (*n* = 3 biological replicates). For each animal, all experiments have been conducted in triplicate. Thus, at least 9 replicates were performed for each experiment. The results were expressed as mean ± standard deviation (S.D.) and were assessed for normal distribution using D’Agostino and Pearson tests. The analyses carried out for statistically assessing the diameter size, fiber alignment and mechanical properties of PLGA fleeces and 3D scaffolds used the two-tailored independent *t* test (GraphPad Prism 9, San Diego, CA, USA). Dataset comparisons concerning the biological analyses were performed by one-way ANOVA multi-comparison tests followed by Tukey post hoc tests (GraphPad Prism 9, San Diego, CA, USA). The values with at least *p* < 0.05 were considered significant.

## 3. Results

### 3.1. Topology and Topography of PLGA 3D Scaffolds Resemble Tendon Macroscopy and Microscopy

**Figure 1:**
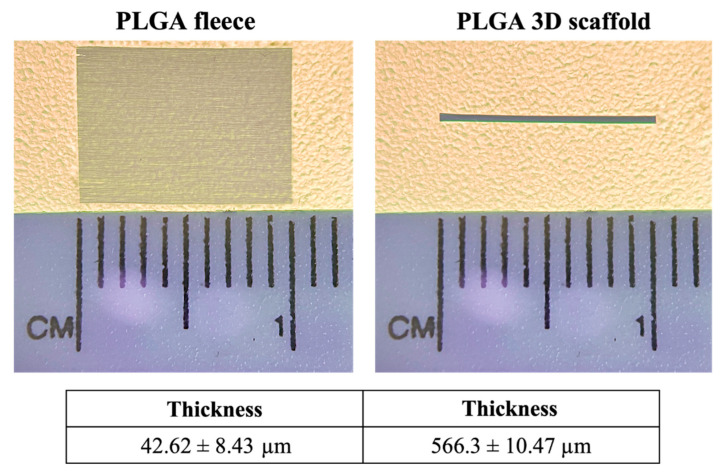
Stereomicroscope observation of PLGA fleeces and 3D scaffolds with their quantified thickness.

The PLGA 3D scaffolds obtained by rolling up the electrospun fleeces showed a thickness of 566.3 ± 10.47 µm, with respect to fleeces that were 42.62 ± 8.43 µm thick (Figure 1).

**Figure 2:**
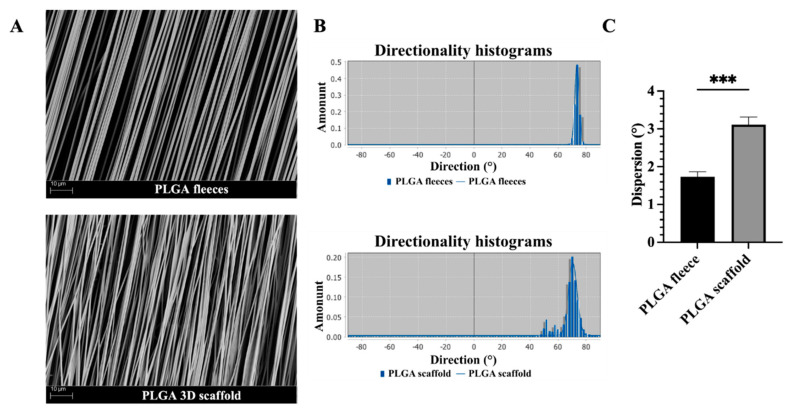
Ultrastructure assessment of electrospun PLGA fleeces and 3D scaffolds. (**A**) SEM micrographs representing the ultrastructural morphology of the produced electrospun PLGA fleeces and 3D scaffolds. All samples showed defect- and bead-free fibers, with high alignment confirming that the rolling-up of the PLGA fleeces does not affect the fiber ultrastructure. (**B**) Fiber orientation of the electrospun PLGA fleeces and scaffolds using Directionality Plugin from ImageJ. Directionality histograms showing the angle distribution (Gaussian curve) of the fibers within the different PLGA fleeces and scaffolds. (**C**) The histogram shows the dispersion values, representing the standard deviation of the Gaussian curves showing the variability in the fiber orientation within the PLGA fleeces and scaffolds. *** Statistically significant between the tested groups (*p* < 0.001).

From an ultrastructure point of view, the produced PLGA fleeces with highly aligned fibers were defect- and bead-free and had an average fiber diameter size of about 1.27 ± 0.11 µm (Figure 2A), similarly to the previously produced PLGA fleeces [16]. Moreover, after being rolled-up, PLGA 3D scaffolds did not show significant alterations in their microfibers’ ultrastructure compared to PLGA fleeces, even if differences in their fiber orientation were observed (Figure 2B). In detail, the PLGA fleeces exhibited the sharpest Gaussian curve with highly oriented fibers represented by the lowest dispersion compared to the PLGA 3D scaffolds (*p* < 0.001), in which the histogram of fibers’ angle distribution became broader (Figure 2B). Even if 3D scaffolds showed a significant variability in dispersion values with respect to PLGA fleeces, in any case most of their fibers were distributed according to a Gaussian curve thus demonstrating in any case a high degree of alignment as shown in Figure 2C.

### 3.2. Mechanical Properties of PLGA 3D Scaffolds Are Enhanced Respect to Fleeces

**Figure 3:**
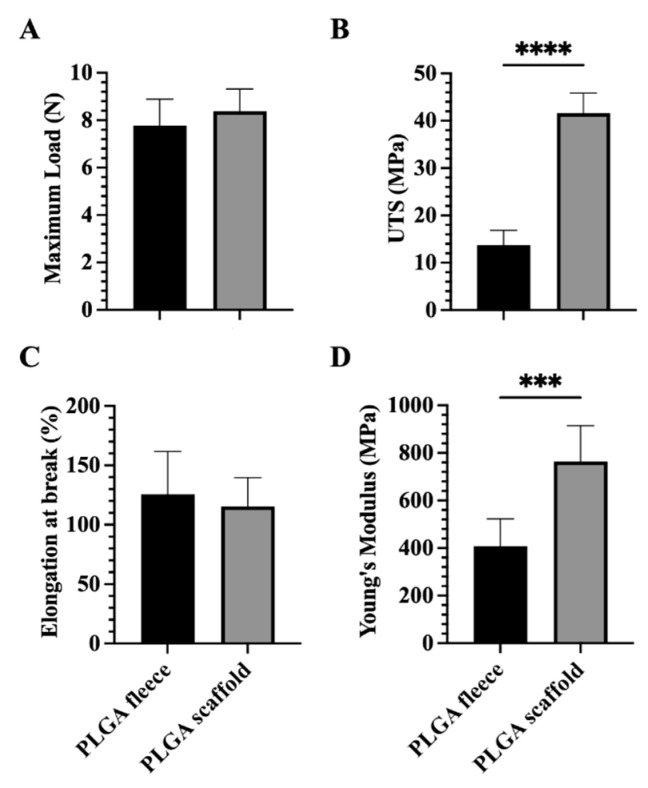
The mechanical tests on the produced PLGA fleeces and scaffolds represented in terms of (**A**) maximum load, (**B**) ultimate tensile strength (UTS), (**C**) elongation at break, and (**D**) Young’s Modulus. *** and **** Statistically significant between different groups (*p* < 0.001 and *p* < 0.0001, respectively).

The variation in the mechanical properties between the PLGA fleeces and rolled-up 3D scaffolds was evaluated in terms of maximum load, Ultimate Tensile Strength (UTS), fracture strain and Young’s modulus (Figure 3).

In detail, no significant difference has been noticed in terms of maximum load between electrospun PLGA fleeces (7.78 ± 1.11 N) and PLGA 3D scaffolds (8.38 ± 0.94 N), even if higher values were obtained in the case of PLGA 3D scaffold (Figure 3A, *p* > 0.05).

The UTS property showed a significant increase in the case of electrospun PLGA 3D scaffolds (41.59 ± 4.27 MPa) compared to PLGA fleeces (13.78 ± 3.08 MPa, Figure 3B, *p* < 0.0001).

No significance has been found in terms of elongation at break property between the different studied groups (Figure 3C, *p* > 0.05). However, slightly higher values were detected in the case of electrospun PLGA fleeces compared to the 3D scaffolds that showed a decrease of about 8% in elongation break values (*p* > 0.05).

The same pattern as UTS was also observed when analyzing Young’s Modulus, where the values for the electrospun PLGA 3D scaffolds (763.8 ± 151 MPa) were significantly higher compared to the PLGA fleeces (406 ± 116.3 MPa, Figure 3D, *p* < 0.001).

### 3.3. PLGA Fiber Alignment and 3-Dimensionality of the Construct Influence AECs’ Morphology

**Figure 4:**
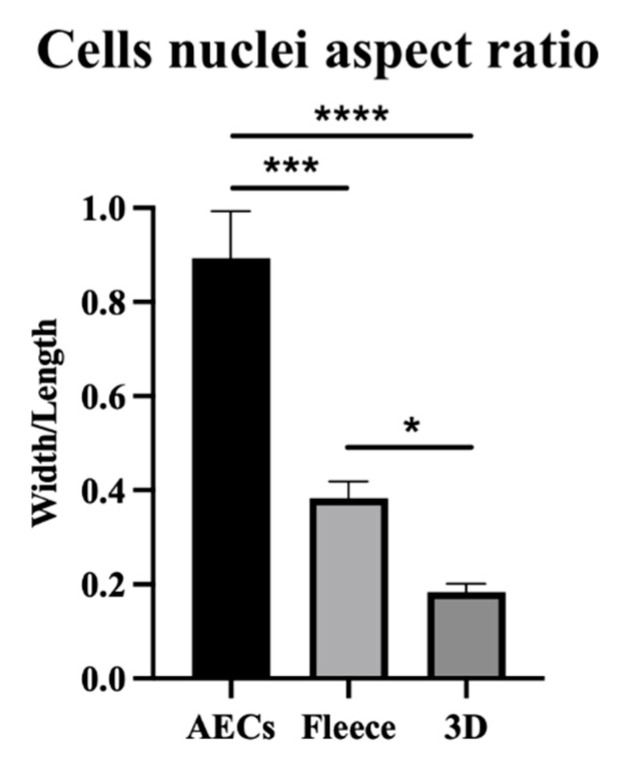
Cells nuclei aspect ratio (width/length) of AECs on PLGA fleeces and 3D scaffolds. AECs on Petri dishes maintain their typical epithelial shape, whereas they acquire an elongated morphology already at 48 h culture on fleeces and 3D scaffolds. *, ***, and **** Statistically significant values between the different studied groups (*p* < 0.05; *p* < 0.001; and *p* < 0.0001, respectively).

Cell nuclei aspect ratio between width and length was quantified in CTR and AECs seeded on PLGA fleeces and 3D scaffolds after 48 h of culture (Figure 4). In detail, CTR nuclei presented a ratio of 0.89 ± 0.1, retaining an AECs typical epithelial cuboidal morphology and ovoidal nuclei (i.e., 1 is perfectly circular). Differently, AECs engineered on as part of both constructs were significantly affected in their morphology, showing a significant decrease in their aspect ratio when seeded on PLGA fleeces (0.38 ± 0.02) and even more when cultured on 3D scaffolds (0.18 ± 0.01) with respect to CTR (*p* < 0.001 and *p* < 0.0001, respectively; Figure 4), acquiring a tenocyte-like morphology. Indeed, cells seeded on 3D scaffolds showed a significant decrease in their cell nuclei aspect ratio with respect to that of PLGA fleeces (*p* < 0.05, Figure 4).

### 3.4. PLGA 3D Scaffolds Possess a Boosted Teno-Inductive Potential on AECs Respect to Fleeces

**Figure 5:**
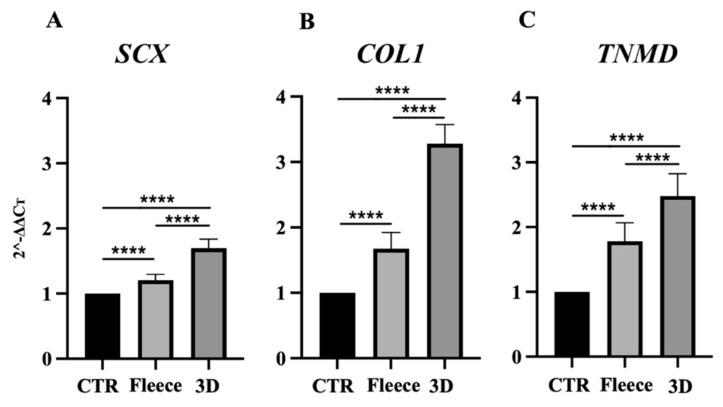
Tendon gene expression profile in AECs seeded on PLGA fleeces and 3D scaffolds. (**A**) *SCX*, (**B**) *COL1*, and (**C**) *TNMD* gene expression after 48 h of culture. AECs on Petri dishes were used as the CTR (AECs at 48 h = 1). **** Statistically significant values between the different studied groups (*p* < 0.0001).

Based on the aspect ratio results, the teno-inductive potential of PLGA 3D scaffolds on AECs was examined by analyzing the gene expression profile of early *SCX* and late *COL1* and *TNMD* tendon markers after 48 h of culture. The expressions of all the analyzed tendon-related genes were significantly higher in AECs cultured on both PLGA constructs with respect to CTR (*p* < 0.0001; Figure 5A–C). In particular, 3D scaffolds exhibited a greater teno-inductive potential, resulting in AECs significant *SCX*, *COL1* and *TNMD* upregulation with respect to cells seeded on fleeces (*p* < 0.0001; Figure 5A–C).

**Figure 6:**
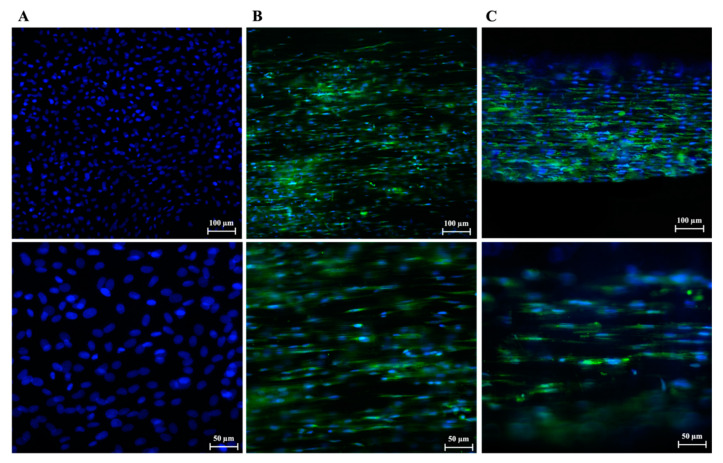
IF images of TNMD protein expression (green fluorescence) in AECs seeded on (**A**) Petri dishes used as CTR, (**B**) PLGA fleeces and (**C**) 3D scaffolds; DAPI (blue fluorescence)-counterstained nuclei.

AECs’ tenogenic differentiation was confirmed by analyzing TNMD protein expression on all tested groups after 48 h of culture. As expected, AECs CTR, which do not normally express TNMD in their cytoplasm, resulted negative to this protein (Figure 6A). Differently, TNMD protein was expressed within AECs engineered on both PLGA fleeces (Figure 6B) and 3D scaffolds (Figure 6C).

### 3.5. PLGA 3D Scaffolds Enhance AECs’ Molecular and Paracrine Immune-Modulatory Function

To gain insights on the effect of both electrospun PLGA highly aligned fiber constructs on the immunomodulatory properties of AECs, the analysis of gene expression profiles of the key anti- (*IL-10*) and pro- (*IL-12*) inflammatory cytokines was performed after 48 h of culture.

**Figure 7:**
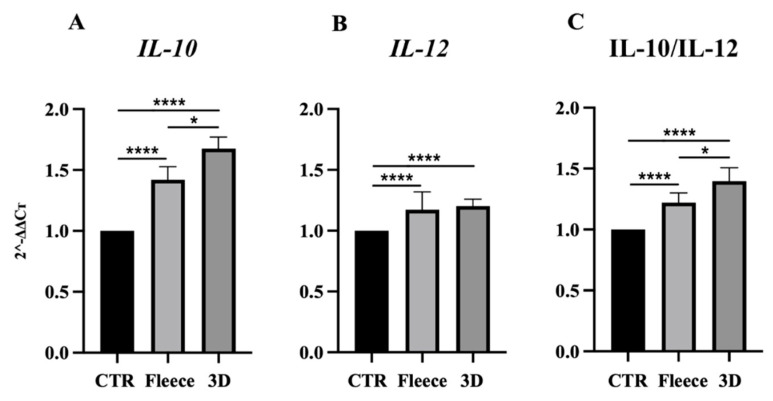
Gene expression profile of (**A**) anti-inflammatory *IL-10* and (**B**) pro-inflammatory *IL-12* cytokines in CTR and AECs seeded on fleeces and on 3D scaffolds after 48 h of culture. AECs after 48 h of culture on Petri dishes was used as the CTR (AECs at 48 h = 1). (**C**) IL-10/IL-12 ratio of the expression levels in CTR and in AECs seeded on fleeces and 3D scaffolds after 48 h. * and **** Statistically significant values between the different studied groups (*p* < 0.05 and *p* < 0.0001, respectively).

As reported in Figure 7A, *IL-10* gene expression increased significantly within both tested electrospun PLGA constructs with respect to CTR (*p* < 0.0001), even if significantly higher values were obtained for 3D scaffolds with respect to PLGA fleeces (*p* < 0.05).

On the contrary, *IL-12* gene expression remained almost constant for all tested samples, with a slight significant increase for the cells seeded on the PLGA fleeces and 3D scaffolds with respect to CTR (*p* < 0.05, Figure 7B).

The increased IL-10/IL-12 ratio in AECs cultured on both electrospun PLGA constructs, showed a preferentially *IL-10* anti-inflammatory gene profile expression rather than an *IL-12* pro-inflammatory one. In detail, cells seeded on both constructs showed a higher IL-10/IL-12 ratio with respect to CTR (*p* < 0.0001, Figure 7C), with significant higher values for 3D scaffolds with respect to PLGA fleeces (*p* < 0.05, Figure 7C).

**Figure 8:**
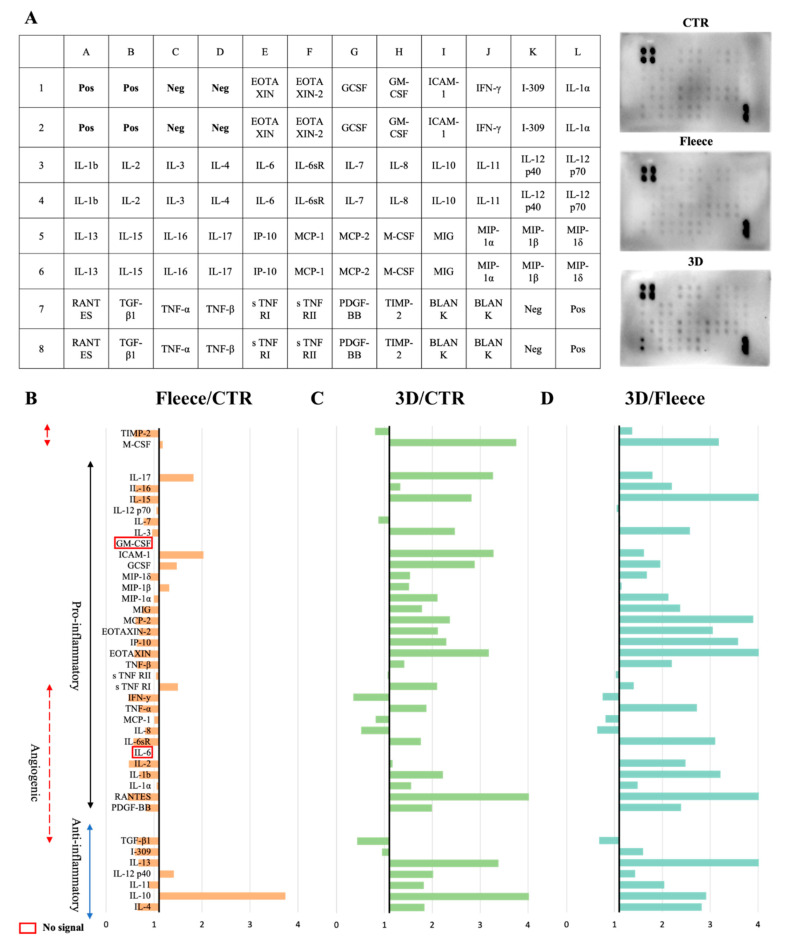
Immunomodulatory factors’ expression profile: anti-inflammatory factors (blue arrow), pro-inflammatory factors (black arrow) and inflammatory molecules with a role in angiogenesis (red dashed arrows). (**A**) Array’s layout and representative images of chemokines detection after 48 h of culture. (**B**) PLGA Fleeces/CTR immunomodulatory factors’ profile expression. CM of AECs seeded on Fleeces showed an almost total downregulation of factors’ release with respect to that of CTR. (**C**) PLGA 3D scaffolds/CTR immunomodulatory factors’ profile expression. CM of AECs seeded on 3D scaffolds showed an almost total enhancement in factors’ release with respect to that of CTR. (**D**) PLGA 3D scaffolds/Fleeces immunomodulatory factors’ profile expression. CM of AECs seeded on 3D scaffolds showed an almost total enhancement in all factors’ release with respect to that of cells cultured on Fleeces.

AECs’ paracrine ability to release immunomodulatory factors in response to their culture on PLGA fleeces or 3D scaffolds was assessed. The CM analyses were performed by using a human inflammation antibody array which recognizes 40 molecules (Figure 8), that were classified into anti-inflammatory and pro-inflammatory factors, some of which have a role in angiogenesis [41]. The protein analysis showed that AECs CTR CM itself contains a basal secretion level of the analyzed immunomodulatory molecules (Figure 8A). Cells seeded on PLGA fleeces showed a significant downregulation of nearly all the immunomodulatory factors with respect to CTR (*p* < 0.05) with the only exception of the anti-inflammatory IL-10 and IL-12 p40 and of the pro-inflammatory sTNF RI, MIP-1β, GCSF, ICAM-1, IL-17 and M-CSF (Figure 8B). On the contrary, for AECs cultured on 3D scaffolds, nearly all the analyzed factors were significantly higher (mostly 1.5-fold more; *p* < 0.05) compared to the corresponding target expression level in CTR, with the only exception of the anti-inflammatory I-309 and TGF-β1, and of the pro-inflammatory IL-8, MCP-1, IFN-γ, IL-7 and the angiogenic TIMP-2 (Figure 8C). Interestingly, AECs cultured on 3D scaffolds significantly expressed higher levels of almost all the analyzed factors compared to the corresponding target protein in AECs seeded on fleeces (*p* < 0.05). However, TGF-β and IL-8, MCP-1 and IFN-γ (Figure 8D) showed lower levels of secretion. Of note, IL-6 and GM-CSF protein contents were not detected in all CM samples probably because antibodies for these two molecules are highly species-specific and thus do not recognize these relative sheep proteins [38].

**Figure 9:**
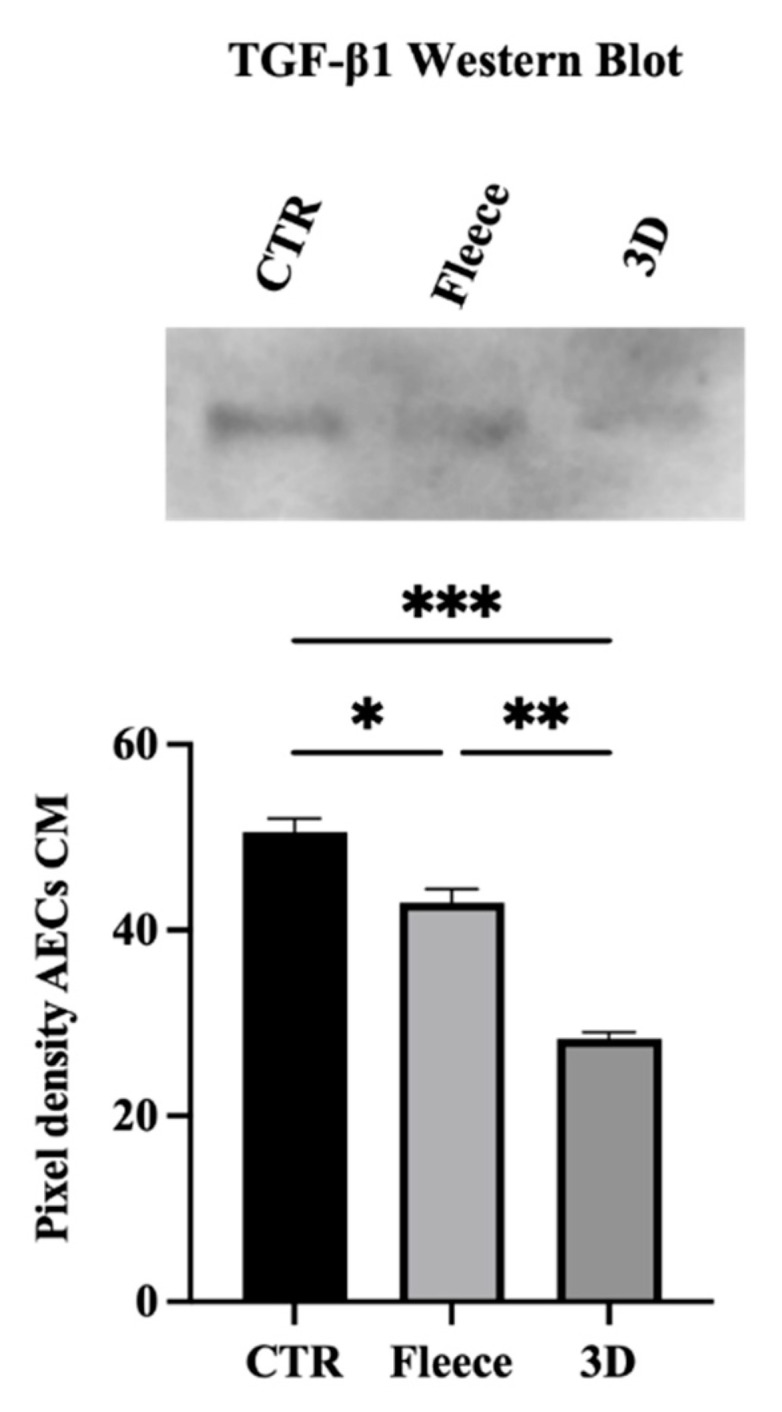
Western Blot confirms TGF-β1 protein CM expression among the different groups as revealed thought the human inflammation antibody array analysis. *, **, and *** are statistically significant values between the different studied groups (*p* < 0.05, *p* < 0.01, and *p* < 0.001, respectively).

As a confirmation of the arrays result, WB analysis of TGF-β1 protein expression (Figure 9) showed that CM obtained from CTR significantly overexpressed the factor with respect to fleeces (*p* < 0.05) and greatly in terms of 3D scaffolds (*p* < 0.001).

**Figure 10:**
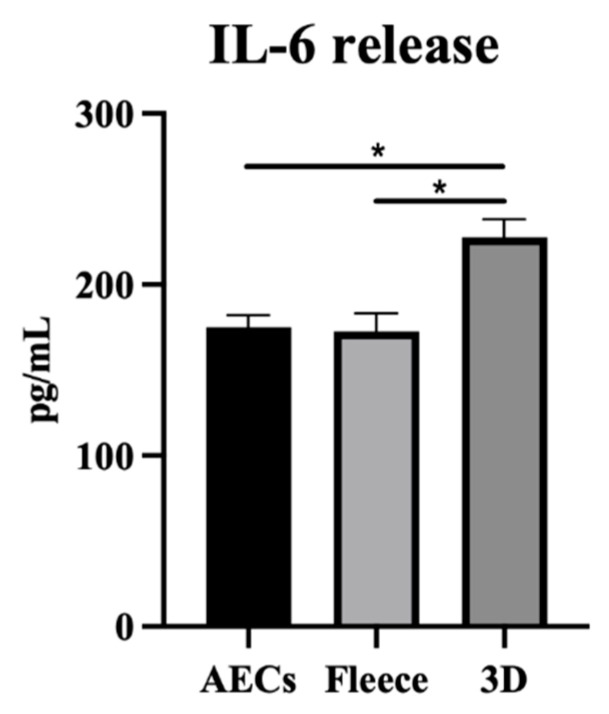
Effect of CM on IL-6 levels released from THP-1 macrophages was evaluated by ELISA. The IL-6 levels in supernatants were analyzed after 24 h treatment through ELISA assay. * Statistically significant values between the different studied groups (*p* < 0.01).

On behalf of the IL-6 and GM-CSF results obtained from the analysis of AECs’ CM and taking advantage of the differential IL-6 expression between human and ovine species, the biological outcome of the obtained ovine CM was further investigated on human THP-1 cells. As shown in Figure 10, CM obtained from PLGA 3D scaffolds induced human macrophages to release significant higher levels of IL-6 with respect to CTR and PLGA Fleeces (*p* < 0.01). Additionally, no significant differences were seen between the IL-6 release profile within CM from AECs CTR and PLGA fleeces (*p* > 0.05; Figure 10).

### 3.6. PLGA 3D Scaffold Greatly Activates AECs’ Mechanosensitive YAP Signaling Pathway over Fleeces

**Figure 11:**
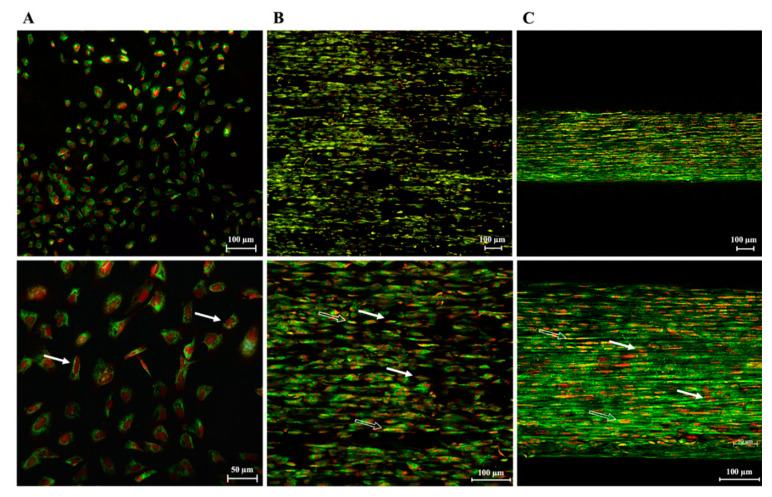
IF analysis of YAP protein expression (green fluorescence) and localization in (**A**) AECs cultured on Petri dishes (CTR) or seeded on PLGA (**B**) fleeces and (**C**) 3D. PI (red fluorescence)-counterstained nuclei. While CTR cells expressed the protein only within the cytoplasm, cells cultured on fleeces and 3D scaffolds localized YAP within the cytoplasm and in the nucleus (yellow nuclei, due to the overlap of red and green fluorescence). Examples of YAP cytoplasmic localization (white arrows) and nuclear translocation (white plain arrows).

**Figure 12:**
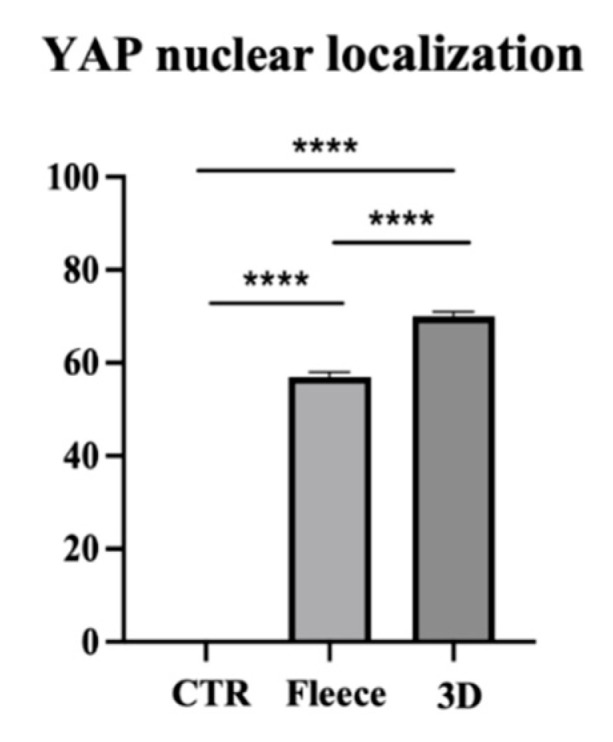
Percentage of YAP nuclear localization after seeding on Petri dishes (CTR), PLGA fleeces and 3D scaffolds. **** Significant difference between the different groups (*p* < 0.0001).

To evaluate the effect of fiber topology and topography on AECs’ biology, YAP, a cell mechanotransducer, has had its protein expression assessed. CTR showed an exclusive uppermost cytoplasmatic localization of YAP protein, confirming its inactivation when cells were cultured under standard conditions (Figure 11A). Quantification of the number of overlapping PI (red fluorescence) and YAP (green fluorescence) nuclei (yellow signal), revealed that in the CTR all the cells displayed YAP protein within their cytoplasm rather than their nucleus (*p* < 0.0001, Figure 12). Instead, cells seeded on PLGA fleeces and 3D scaffolds showed YAP positivity both in the cytoplasm and in the nuclei, demonstrating a nuclear translocation of the protein, evident from the yellow color of the nuclei (Figure 11B,C). In detail, cells on PLGA fleeces and 3D scaffolds displayed a significant and predominant YAP nuclear positivity with respect to CTR (57% and 70% respectively vs. 0%; *p* < 0.0001; Figure 12). Moreover, PLGA 3D scaffolds exhibited a significant nuclear localization with respect to fleeces (70% vs. 57%; *p* < 0.0001; Figure 12).

## 4. Discussion

The synergic use of cells and materials has emerged as an important approach in TE, in which understanding the interaction between cells, materials and their topographical and topological properties becomes essential to develop functional scaffolds for regenerative medicine. Biomaterials should be developed to provide the most suitable microenvironment, capable of guiding cells’ genotype, phenotype and paracrine function [6].

In this context, a key factor to be considered when designing a scaffold for tendon TE is its biomimicry in terms of 3D morphology and ultrastructure to resemble the structural organization and mechanical properties of the native tissue [7,8]. To reach this goal, in this study, 3D tendon-like scaffolds, made by rolling up electrospun PLGA fleeces with axially aligned microfibers [15,16,17], were fabricated. This approach allowed 3D scaffolds to resemble tendon collagen fibers alignment and, in terms of topology, the diameter of tissue fascicles (100–500 µm) that contain collagen microfibers [42,43]. Accordingly, the 3D scaffolds’ average diameter size was within the range of that of tendon collagen fibers (1–20 µm) where tenocytes reside [44,45,46,47]. Similarly to what was previously observed in PLGA fleeces [15,16,17,34], SEM analyses demonstrated that PLGA 3D scaffolds exhibited a good fiber alignment and arrangement, which could be considered desirable features to mimic tendon morphology [48,49,50].

The produced PLGA 3D scaffolds exhibited better mechanical properties compared to PLGA fleeces. In particular, in PLGA 3D scaffolds, there was an increase of about 68%, 7% and 45% in UTS, maximum load, and Young’s modulus, respectively. On the contrary, the slight decrease in elongation at the break within 3D scaffolds (about 8%) might be attributed to the high resistance to the uniaxial tension exhibited by the aligned fibers when they grouped together. The mechanical properties of the electrospun 3D scaffolds closely resemble the mechanical properties of human patellar, rotator and Achilles tendons in terms of UTS and Young’s modulus [2]. Thus, the obtained results and the high mechanical performance of the 3D PLGA scaffolds could make them ideal for surgical applications and considered as crucial factors to be considered in tendon TE.

In accordance to literature [26,51,52], these 3D scaffolds showed themselves to possess a great impact on AECs’ biology and function. In particular, the comparative biological performance of the fabricated electrospun PLGA 3D scaffolds and fleeces was evaluated on AECs after being engineered up to 48 h of culture on which teno- and immune-inductive potentials have been assessed without adding any tenogenic differentiation and immunostimulatory factors. This time span was selected according to previous studies in which it was demonstrated that AECs engineered on highly aligned constructs differentiated towards the tenogenic lineage and modulated *IL-10* and *IL-12* gene expression profile already after 48 h of culture [15,16,17]. As a support, it has been evidenced that 48 h represents the initial time contact between the scaffold and the host tissue, as well as, the early inflammatory phase of tendon healing [26,34,53,54].

The obtained results, according to previously published studies, confirmed that fiber alignment is essential in guiding cell organization and morphology alongside the fiber arrangement [15,25,55,56,57,58,59]. In this context, the aligned topography of PLGA fibers influenced AECs morphology by changing their phenotype towards a tenocyte-like one confirmed by a significant decrease in cellular aspect ratio compared to native AECs (CTR) which retained a native polygonal morphology, as demonstrated previously [16]. However, 3D scaffolds displayed a boosted effect on modulating AECs’ phenotype represented by a significantly inferior ratio with respect to the CTR and interestingly also with respect to fleeces. Accordingly, it was demonstrated that PLGA 3D scaffolds boosted AECs trans-differentiation toward the tenogenic lineage, acquiring a tenocyte-like genotype and phenotype, compared to fleeces. Indeed, the seeded cells on the 3D constructs significantly up regulated tendon-related genes (*SCX*, *COL1*, and *TNMD*) and TNMD protein cytoplasm expression already after 48 h of culture. *SCX* is a neotendon formation marker implicated in fetal tendon formation, and in adult tendons it is involved in their homeostasis as a co-activator of other tendon-related genes like *COL1* and *TNMD* [60,61,62]. TNMD, a tendon differentiation and mature tendon marker, is required for the creation of collagen networks and tendon organization [60,63]. SCX and TNMD are required for proper COL1 matrix assembly and structural integrity [39], while COL1 is the most abundant protein in a native tendon [60,64]. Interestingly, the obtained data strongly suggest that not only the fiber alignment stimulate AECs’ tenodifferentiation, as already demonstrated with PLGA highly aligned fleeces [15,16,17], but also the construct topology (i.e., its 3-dimensionality) is crucial to enhance even more their differentiation towards the tenogenic lineage recreating a more favorable tenogenic microenvironment.

It has been recently proven that scaffolds can positively contribute in modulating the inflammatory response of the native tissue and stimulate its regeneration [26]. Immunomodulation by biomaterials and fiber alignment affects macrophage polarization from inflammatory (M1) to anti-inflammatory/pro-regenerative (M2) phenotypes in the host tissue, or even enhances stem cell immunomodulatory function especially in the first 48 h [26]. In the present work, after 48 h of culture, PLGA fleeces and 3D scaffolds showed a significant upregulation of the anti-inflammatory cytokine *IL-10* gene expression with respect to fleeces and even more with respect to the CTR, while the pro-inflammatory cytokine *IL-12* remained at their basal levels within all tested groups. Thus, AECs engineered on the PLGA constructs, displayed a positive IL-10/IL-12 ratio, that was significantly higher with respect to CTR, confirming the findings of a positive immunomodulatory role of the fiber alignment [16] but also demonstrating the higher immune-inductive potential of 3D scaffold topology with respect to fleeces. Moreover, also in this case, 3D scaffolds showed to significantly increase the anti-inflammatory potential on AECs with respect to fleeces. Indeed, the high IL-10/IL-12 ratio, could support a rapid transition of AECs from the inflammatory to the reparative phase denoting a possible role in shifting M1 macrophages in M2 phenotype [36,65].

3D scaffolds also potentiated the paracrine function of AECs, which per se releases immunomodulatory molecules. Indeed, for the first time, it was evidenced that CM obtained from cells seeded on fleeces and greatly on 3D scaffolds and expressed a significant increase in anti-inflammatory molecules, amongst which IL-10 increased with respect to the CTR, confirming the results obtained from mRNA analysis. At the same time, CM collected from AECs engineered on 3D scaffolds showed a CM enrichment of immunomodulatory factors compared to CTR. Looking into these immunomodulatory molecules, many of them exert a positive role in the field of tendon regeneration. For example, PDGF-BB, RANTES, IL-lα, IL-1β, IL-2, IL-6sR and TNF-α, generally considered pro-inflammatory, exhibit a role in stimulating angiogenesis [41], and an overproduction of these molecules by the cells seeded on 3D scaffolds might suggest a positive role for the construct in tendon regeneration [66]. In particular, PDGF-BB is a crucial growth factor for increasing tenocyte proliferation and matrix remodeling, especially during the first phases of tendon healing [67,68,69]. Moreover, also the upregulated pro-inflammatory IP-10, RANTES, MIP-1α and GCSF molecules found in the 3D scaffolds’ CM, can be considered as rising key factors for tendon repair and healing. In detail, Stålman et al. suggested IP-10, RANTES, MIP-1α as chemokines with a role in promoting vascular and neuronal mediators essential for the tendon healing process [70]. Moreover, GCSF was investigated by Ross et al., who found an increase in local cellularity after rotator cuff repair thorough GCSF treatment [71]. Knowing that TGF-β1 is secreted by AECs [19,38], its release in CM was evaluated by WB, confirming the array results.

Since the array analysis alone is not predictive of the cells’ immunomodulatory activity, the biological test on human THP-1-activated macrophages was crucial for assessing the potential immunomodulatory effect of AECs’ CM. This analysis allowed us to demonstrate firstly that AECs can dialogue in a xeno-system, promoting macrophages activity by secreting specific immunomodulatory molecules. The important naïve immunomodulatory action of released factors in CM from untreated AECs was demonstrated, supporting their immunogenic role when they are transplanted in tissue lesions favoring its regeneration process [20,21]. In this regard, the CM obtained from the different culture conditions were able to induce on THP-1-activated macrophages the human-specific IL-6 release. However, 3D scaffolds’ CM significantly increased IL-6 secretion despite those of CTR and PLGA fleeces. It has been demonstrated that this cytokine, which is commonly considered as a pro-inflammatory one, is a key molecule in tendon healing. Indeed, in a study conducted by Ackermann et al. showed that tendon repair is characterized by an upregulation of IL-6 during the resolving inflammatory phase [72], which raises to play an important role in tendon healing [73,74].

The obtained results highlight the ability of PLGA 3D scaffolds to greatly influence AECs’ immunomodulatory properties, recreating a favorable environment, necessary for dictating positive host responses and beneficial for tissue healing.

Scaffolds’ topography and topology greatly induced AECs’ tenodifferentiation and immunomodulation that are mediated by YAP mechanotransducer andinvolved in both responses as described in the literature [26,27,29,51]. Indeed, higher YAP nuclear translocation was observed within cells engineered on 3D scaffolds with respect to those seeded on fleeces. On the contrary, AECs CTR displayed exclusively a cytoplasmatic localization of YAP protein. Moreover, these results demonstrate for the first time that AECs activated the signal transduction YAP pathway when engineered on PLGA constructs.

Altogether, the obtained results suggest that biomaterials’ topography and topology are essential in modulating the implant–tissue response. However, the more instructive performance of PLGA 3D scaffolds over PLGA fleeces, both equipped with aligned fibers, have highlighted the importance of the 3D milieu provided by the scaffold, which probably promotes a better cell–cell interaction with respect to less stratified cultures as when culturing the cells on fleeces. It is conceivable that PLGA 3D scaffolds mimicking the tendon microarchitecture instruct the seeded AECs by recreating the biological microenvironment of the native tendon tissue, thus boosting the release from AECs of key molecules crucial for tendon healing. These promising results must be confirmed in vivo in preclinical tendon injury models in order to evaluate the regenerative potential of the fabricated 3D scaffolds. Moreover, in the future, this 3D scaffold could be used to develop a complex device composed of multiple 3D units (i.e., several fascicles) that mimics a full tendon to be applied in human and animal patients.

## 5. Conclusions

In this study, it has been demonstrated that rolling-up the PLGA fleeces is an easy way to obtain 3D tendon-like scaffolds with a good fiber alignment and enhanced mechanical properties. In addition, through a mechanosensing mechanism, PLGA 3D scaffolds boosted AECs’ tenodifferentation and immunomodulatory properties by creating a tailored microenvironment, closer to that of the native tendon, crucial to potentially promoting positive host responses and tissue regeneration. The encouraging results strongly suggest that the fabricated biomimetic PLGA 3D scaffolds are potential candidates for tendon regeneration suitable for surgical purposes.

## Figures and Tables

**Table 1:** Details on primer sequences.

Gene	Forward Primer (5′ to 3′)	Reverse Primer (5′ to 3′)	Product Size (bp)
*GAPDH*	CCTGCACCACCAACTGCTTG	TTGAGCTCAGGGATGACCTTG	224
*IL-10*	CCAGGATGGTGACTCGACTAG	TGGCTCTGCTCTCCCAGAAC	76
*IL-12b*	ACAAAGGAGGCGAGGTTCTG	CTGTGGTCCATGCTGACCTT	283
*TNMD*	TGGTGAAGACCTTCACTTTCC	TTAAACCCTCCCCAGCATGC	352
*SCX*	AACAGCGTGAACACGGCTTTC	TTTCTCTGGTTGCTGAGGCAG	299
*COL1*	CGTGATCTGCGACGAACTTAA	GTCCAGGAAGTCCAGGTTGT	212

Primers used in Citeroni et al. [14] and El Khatib et al. [34].

## Data Availability

The data presented in this study are available in the article.
